# Bilirubin inhibits the anticancer activity of sorafenib by blocking MCL-1 degradation in hepatocellular carcinoma cells

**DOI:** 10.20892/j.issn.2095-3941.2021.0598

**Published:** 2022-05-24

**Authors:** Leyi Yao, Qian Zhao, Ding Yan, Ziying Lei, Yali Hao, Jinghong Chen, Qian Xue, Xiaofen Li, Qingtian Huang, Daolin Tang, Q. Ping Dou, Xin Chen, Jinbao Liu

**Affiliations:** 1 Affiliated Cancer Hospital & Institute of Guangzhou Medical University, Guangzhou 510095, China; 2 Guangzhou Municipal and Guangdong Provincial Key Laboratory of Protein Modification and Degradation, State Key Laboratory of Respiratory Disease, School of Basic Medical Sciences, Guangzhou Medical University, Guangzhou 511436, China; 3 Institute of Digestive Disease of Guangzhou Medical University, The Sixth Affiliated Hospital of Guangzhou Medical University, Qingyuan People’s Hospital, Qingyuan 511518, China; 4 School of Public Health, Guangzhou Medical University, Guangzhou, 511436, China; 5 Department of Surgery, UT Southwestern Medical Center, Dallas, TX 75390, USA; 6 Barbara Ann Karmanos Cancer Institute and Departments of Oncology, Pharmacology & Pathology, School of Medicine, Wayne State University, Detroit, MI 48201, USA

**Keywords:** Apoptosis, bilirubin, hepatocellular carcinoma, MCL-1, sorafenib

## Abstract

**Objective:**

Sorafenib is a first-line drug for advanced hepatocellular carcinoma (HCC). Unfortunately, most patients with HCC do not respond to sorafenib, mainly because of the frequent development of drug resistance. Bilirubin is an end metabolite of heme catabolism and an indicator of liver function, but its direct role in regulating the anticancer activity of sorafenib in HCC cells is unclear. In the current study, we aimed to investigate the mechanism of action of bilirubin in sorafenib-mediated tumor suppression in HCC.

**Methods:**

A retrospective observational cohort of 100 patients receiving sorafenib was conducted to evaluate the potential role of bilirubin in predicting the prognosis of patients with HCC. Human HCC cell lines were treated with sorafenib in the absence or presence of bilirubin, and cell proliferation, apoptosis, and signaling pathways were assayed. The antagonistic effect of bilirubin toward sorafenib was assessed in nude mice bearing HCC xenografts.

**Results:**

Serum levels of bilirubin (including total, direct, and indirect bilirubin) negatively correlated with the overall survival of patients with HCC treated with sorafenib (*P* < 0.05). Both *in vitro* and *in vivo* analyses demonstrated that bilirubin significantly abrogated sorafenib-mediated proliferation inhibition and apoptosis induction in HCC cells (*P* < 0.05). Mechanically, bilirubin inhibited sorafenib-induced activation of GSK-3β and subsequent downstream MCL-1 degradation.

**Conclusions:**

Our study provides experimental evidence of the antagonistic effect of bilirubin toward sorafenib-mediated anticancer activity in HCC, and it suggests that bilirubin could be used to predict the efficacy of sorafenib treatment.

## Introduction

Hepatocellular carcinoma (HCC), the most common type of liver cancer, is a leading cause of cancer-associated deaths worldwide^1,2^. Approximately 632,320 cases and 577,522 deaths in men, and 273,357 cases and 252,658 deaths in women were estimated worldwide in 2020^3^. From 2007 to 2016, the rate of increase in liver cancer mortality was the fastest among all cancers^1^. Worldwide, East and Southeast Asia have the highest rates of HCC, and China alone accounts for 50% of the total global HCC cases and deaths^4,5^. Therefore, attention should be paid to the prevention, detection, diagnosis, and treatment of HCC^6^.

Sorafenib is an oral multi-kinase inhibitor that blocks the activity of platelet-derived growth factor receptor (PDGFR), vascular endothelial growth factor receptor (VEGFR), Fms related receptor tyrosine kinase 3 (FLT3), and c-Kit, as well as the RAF/MEK/ERK pathway^7^. Sorafenib was approved for the treatment of advanced HCC by the U.S. Food and Drug Administration as the first-line systemic therapy^8,9^. Although sorafenib prolongs the survival of patients with advanced HCC, it causes clear adverse reactions^10–12^. Moreover, many patients develop acquired resistance to sorafenib. Therefore, reliable predictive markers or methods are urgently needed to identify patients likely to be susceptible to sorafenib and to exclude those unlikely to respond.

Levels of some plasma proteins, such as vascular endothelial growth factor A (VEGFA), angiopoietin-2 (Ang-2), and insulin like growth factor 1 (IGF1), have been reported to predict the outcomes of sorafenib-based therapy^13,14^. Liver function should also be considered, because chronic liver disease is associated with HCC in most patients. In this regard, according to 2 phase-III studies of sorafenib, most patients have Child-Pugh A cirrhosis, which can affect the efficacy of sorafenib^15^. Indeed, the GIDEON study has reported that in patients receiving sorafenib therapy, those with Child-Pugh A have a significantly longer median OS than those with Child-Pugh B or Child-Pugh C, at 13.6 moths, 5.2 months, and 2.6 months, respectively^16^. However, the mechanism of liver function in the failure of sorafenib treatment remains unclear.

Bilirubin is the end metabolite of heme catabolism by the enzyme heme oxygenase. There are 2 forms of serum total bilirubin: indirect bilirubin and direct bilirubin. Indirect bilirubin accounts for as much as 95% of total bilirubin, which is converted to direct bilirubin by the hepatic enzyme UDP glucuronosyltransferase family 1 member A1 (UGT1A1)^17^. Of note, use of the albumin-bilirubin (ALBI) grade is recommended to assess liver function in patients with HCC, thus providing a simple and objective liver function assessment method, in comparison to the Child-Pugh system^18^. Indeed, the ALBI grade can successfully predict the outcomes of patients receiving sorafenib therapy^19–22^. Although prior studies have indicated their potential prognostic value regarding liver function in HCC, whether bilirubin levels could be used as an independent biomarker to predict sorafenib response remains to be verified. We hypothesized that bilirubin plays a direct role in determining susceptibility to sorafenib treatment in patients with HCC. In the current study, we discovered that bilirubin inhibits the anticancer activity of sorafenib through preventing GSK-3β activation-mediated MCL-1 degradation both *in vitro* and *in vivo*.

## Materials and methods

### Reagents

Bilirubin (cat. No. B4126) was purchased from Sigma-Aldrich (St. Louis, MO, USA). Sorafenib (cat. No. HY-10201) was purchased from MedChemExpress (MJ, USA). TWS119 (cat. No. S1590) was purchased from Selleck Chem (TX, USA). Primary antibodies to the following were purchased from Cell Signaling Technology (MA, USA): PARP (#9542, RRID:AB_2160739), caspase 3 (#9662), cleaved caspase 3 (#9661), caspase 8 (#9746), cleaved caspase 8 (#9496), caspase 9 (#9508), Bax (#5023), BCL-2 (#4223), Bim (#2933), MCL-1 (#39224 or #94296), K48 (#8081), α-Tubulin (#2125), phospho-GSK-3β (Ser9) (#9323 or #5558), GSK3β (#12456), Akt (#4691), phospho-Akt (Ser473) (#4060), phospho-JNK (#9255), JNK (#9252), phospho-p38 (#4511), p38 (#8690), Ki-67(#9449), phospho-ERK1/2 (Thr202/Tyr204) (#4370), and ERK1/2 (#4695).

### Patients and ethics statement

We conducted a retrospective study of 100 patients with HCC (88 men and 12 women) who were hospitalized and treated with sorafenib at the Affiliated Cancer Hospital & Institute of Guangzhou Medical University (between January 2009 and December 2016). Diagnosis of liver cancer was confirmed by qualified physicians using standard criteria^23^. The ages of all patients ranged from 18 to 80 years. The study excluded patients who were diagnosed with (or suspected of having) other primary malignant tumors, severe heart disease, arrhythmia, and concurrent infection, and women who were pregnant or breastfeeding. In accordance with the precepts established by the Helsinki Declaration, written informed consent was obtained from all participants, and the study was approved by the Ethical Committee of the Affiliated Cancer Hospital & Institute of Guangzhou Medical University (Approval No. 2021003).

### Cell lines and cell culture conditions

The human hepatoma cell lines HepG2 (American Type Culture Collection, HB-8065) and Hep3B (American Type Culture Collection, HB-8064) were grown in RPMI-1640 medium (Thermo Fisher Scientific, cat. No. C11875500BT) and DMEM (Thermo Fisher Scientific, cat. No. C11995500BT), respectively, supplemented with 10% fetal bovine serum (Biological Industries, cat. No. 04-001-1ACS) at 37 °C and 5% CO_2_. All cells were mycoplasma-free and authenticated with short tandem repeat DNA profiling analysis.

### Western blot analysis

Hepatoma cells were exposed to the indicated treatments, and whole cell protein extracts were prepared with cell lysis buffer (Cell Signaling Technology, cat. No. 9803) containing protease inhibitors and phosphatase inhibitors. The supernatants of the whole cell lysates were collected by centrifugation at 12,000 rpm for 10 min at 4 °C, and the protein concentration was quantified with BCA kits (Thermo Fisher Scientific). Protein samples were prepared with sample loading buffer, then separated by SDS-PAGE and subjected to protein transfer to PVDF membranes. The membranes were blocked with 5% non-fat milk powder in 1× TBST for 1 h. Subsequently, membranes were washed 4 times with 1× TBST for (5 min each). The membranes were incubated overnight at 4 °C with primary antibodies at a 1:1000 dilution, then washed with 1× TBST 4 times. Then secondary HRP-conjugated antibodies were used at a dilution of 1:5000. After incubation for 1 h, the membranes were washed 4 times with 1× TBST (5 min each). Finally, protein bands were detected with ECL detection reagents and exposed to X-ray films (Kodak, Japan). Protein expression levels were quantified in Image J software (RRID:SCR_003070).

### Cell viability assays with MTS

Cell viability was measured with a Cell Titer 96^®^ Aqueous one solution cell proliferation assay kit (Promega, cat. No. G111). HepG2 Cells were seeded in 96-well plates with 100 µL RPMI-1640 complete medium, whereas Hep3B cells were suspended in DMEM with 10% FBS. After 24 h cell culturing, the complete medium was replaced with serum-free medium for starvation overnight. Cells were treated with bilirubin and sorafenib for 24 h. Subsequently, 20 µL MTS was directly added to the wells, and cells were incubated for an additional 2 h. The optical density was determined according to the absorbance measured with a microplate reader (Thermo Fisher Scientific) at a wavelength of 490 nm.

### Cell viability assays with crystal violet staining

Crystal violet assays were performed as previously reported^24^. HepG2 and Hep3B human hepatoma cells were seeded in 96-well plates and incubated for 18–24 h, then starved overnight. Defined concentrations of sorafenib and bilirubin in 100 µL serum-free medium were added to the wells. After a 24 h treatment, the medium with drugs was aspirated, and the plates were gently washed twice with water. The plates were inverted on filter paper and tapped gently to remove any remaining liquid. Cells in each well were stained with 50 µL of 0.5% crystal violet staining solution (0.5% crystal violet in 20% methanol), then incubated for 20 min at room temperature. Subsequently, plates were washed twice with tap water. Plates were air dried at room temperature, and 200 µL methanol was added and incubated 30 min. The optical density was determined with a microplate reader (Thermo Fisher Scientific) according to the absorbance at a wavelength of 570 nm. The measured values were compared with those of the control group, which were normalized to 100%. For statistical analysis, 3 independent experiments for each cell line were performed.

### Cell death assays

An Annexin V-FITC/PI apoptosis detection kit (Sungene Biotech, cat. No. AO2001-02P-H) was used to detect cell apoptosis by flow cytometry as described previously^25^. Hepatoma cells were seeded in 6-well plates. Then the cells were cultured for 24 h in complete medium. Afterward, the cells were incubated with sorafenib and bilirubin for 24 h. The medium was discarded, and the cells were washed with PBS twice. After trypsinization for 1 min, cells were collected and resuspended in binding buffer, and PI and Annexin V-FITC were added according to the manufacturer’s protocol, and incubated for 15 min in the dark. Finally, apoptotic cells were detected with a flow cytometer (Becton Dickinson Biosciences).

### Colony formation assays

Colony formation assays were used to detect the differences in cell proliferation ability after drug treatment. The assays were performed as described previously^26^. HepG2 and Hep3B cells were exposed to single or combination treatment with bilirubin and sorafenib in serum-free medium for 24 h. Then the HepG2 and Hep3B cells were digested and seeded in 6-well plates supplemented with 10% FBS RPMI-1640 medium and DMEM, respectively. Cells were cultured in an atmosphere of 5% CO_2_ at 37 °C for 10–14 days. After being washed with PBS, cells in 6-well plates were fixed in 4% polyformaldehyde for 15 min at 4 °C. Then crystal violet solution (0.5%) was used to stain cells for 20 min. Colonies larger than 60 µm were counted, and the experiments were conducted in triplicate.

### EdU staining

Staining with 5-ethynyl-2′-deoxyuridine (EdU) was performed with a Cell-Light™ EdU Apollo^®^488 In vitro Imaging Kit (RiboBio, cat. No. C10310-3) at a final concentration of 50 µM. Cells were seeded in a chamber slide and cultured for 18–24 h. Cells were exposed to bilirubin and sorafenib (single treatment or in combination) for 12 h. EdU was suspended in complete medium and added to the chamber slide. After incubation for 4 h, the cells were washed with PBS twice. Then 4% paraformaldehyde was used to fix cells for 15 min. Staining was performed according to the manufacturer’s protocol. Then DAPI (Abcam, Cambridge, UK) was used to stain DNA. Pictures of EdU-labeled cells were captured with an Olympus fluorescence microscope, and 3 independent experiments were performed.

### Quantitative reverse transcription PCR (RT-qPCR)

After cell treatment with bilirubin and/or sorafenib for 6 h, total RNA was extracted from cells with TRIzol reagent (Invitrogen, Shanghai, China) according to the manufacturer’s instructions, and the concentration of total RNA was measured with a NanoDrop 2000 spectrophotometer (Thermo Fisher Scientific). Subsequently, reverse transcription (RT) reactions were performed with a Prime Script RT reagent Kit with gDNA Eraser (Takara, Guangzhou, China). After the complementary DNA was obtained, Real-time quantitative PCR (RT-qPCR) analysis was performed with SYBR Premix Ex TaqTM (Takara, Guangzhou, China) according to the manufacturer’s protocol, and each sample was analyzed in triplicate. The amount of gene amplification was detected with a StepOne Plus™ Real-Time PCR System (Applied Biosystems, USA). The PCR conditions were as follows: 95 °C for 30 s, then 40 PCR cycles at 95 °C for 5 s and then 60 °C for 30 s. After the amplification cycles, the melting curve reaction was performed. The relative levels of gene expression (mRNA) were calculated with the 2^−ΔΔCt^ method^27^ and genes were normalized to the 18S nuclear ribosomal RNA small subunit. The primer sequences were as follows:

MCL-1 forward, 5’-TGCTTCGGAAACTGGACATCA-3’;

MCL-1 reverse, 5’-TAGCCACAAAGGCACCAAAAG-3;

18S forward, 5’-CCCAGTAAGTGCGGGTCATAA-3;

18S reverse, 5’-CCGAGGGCCTCACTAAACC-3.

### Immunofluorescence assays

Cells were seeded in a chamber slide and incubated for 18–24 h, then treated with bilirubin and sorafenib, or their combination, for the indicated times. The medium was discarded, and the cells were washed with PBS. Next, 4% paraformaldehyde was added to fix the cells for 15 min at 4 °C. After being washed with PBS 3 times, cells were permeabilized with 200 µL 0.5% Triton X-100 for 5 min. Subsequently, the cells were blocked with 10% FBS in PBS for 1 h at room temperature, then incubated with primary antibodies (diluted in 10% FBS blocking solution) overnight at 4 °C and subsequently washed with PBS. Finally, the secondary antibodies were incubated at a 1:400 dilution in blocking solution for 1 h at room temperature. The cells were rinsed with PBS 3 times/5 min, and fluorescent mounting medium with DAPI (Abcam, Cambridge, UK) was used. Images were captured with a confocal microscope (SP8, Leica) 3 independent times.

### Flow cytometry analysis of intracellular protein

To stain intracellular proteins, we fixed cells in pre-chilled 4% paraformaldehyde, permeabilized them with 90% methanol, and then washed them with PBS. The cells were then stained sequentially with primary antibodies (anti-MCL-1, CST #94296; anti-p-GSK-3β, CST #5558) for 1 h and secondary antibodies (Abcam #ab150077) for 0.5 h. Finally, the stained cells were subjected to flow cytometry analysis (Becton Dickinson Biosciences).

### Immunoprecipitation assays

Immunoprecipitation was performed according to the procedure reported in a previous study^28^. Briefly, anti-MCL-1 magnetic beads were prepared through anti-MCL-1 antibody bio-conjugation [with Protein A/G Magnetic Beads (Thermo Fisher Scientific)] at 4 °C under stirring for 18 h. Cell lysates were obtained after single or co-treatment with bilirubin and sorafenib for 24 h, in the presence of 10 µM MG132 for the final 6 h. Cell lysates were then incubated with anti-MCL-1 magnetic beads at room temperature for 2 h. The immune-complexes were subjected to Western blot analysis as previously described.

### RNA interference

RNA interference assays were performed according to a previous report^29^. Briefly, HCC cells were seeded in 6-well plates and cultured for 18–24 h. Then siRNAs to human MCL-1 or control siRNA (20 nM), Lipofectamine (Invitrogen), and RPMI Opti-MEM (Gibco) were mixed and added to each group. After 24 h, the cells were treated with bilirubin and/or sorafenib for an additional 24 h. The siRNAs were designed and synthesized by RiboBio company (Guangzhou, China). The target sequences of MCL-1 siRNA were as follows: MCL-1 siRNA-1: 5′-CCGCCGAATTCATTAATTT-3′; MCL-1 siRNA-2:5′-GGACTTTTAGATTTAGTGA-3′.

### Nude mouse xenograft model

Nude male BALB/c mice (RRID:IMSR_ORNL:BALB/cRl) were purchased from the Guangdong Animal Center. The use and care of experimental animals were approved by the Institutional Animal Care and Use Committee of Guangzhou Medical University (Approval No. GY2020-094). Mice were kept under inspection for 3 days, then were housed in a barrier facility at the Guangzhou Medical University Animal Center. Adequate food and water were supplied. Approximately 1 × 10^6^ HepG2 cells were carefully implanted subcutaneously on the right flank of each mouse. The mice were divided into 4 groups randomly after 1 week. No blinding was used during the experiment. Each group was treated daily with vehicle, intraperitoneal injection of bilirubin (20 mg/kg/d), and/or oral administration of sorafenib (60 mg/kg/d) for 23 days. Tumor size was evaluated every 2 days, and tumor volumes were calculated with the following formula: *a^2^* × *b* × 0.5, where *a* is the smallest diameter, and *b* is the diameter perpendicular to *a*.

### Immunohistochemical staining

Immunohistochemistry was conducted as follows. Xenograft tumors were embedded in paraffin and sectioned. Sections were deparaffinized and hydrated in alcohol. Then the specimens were washed in tap water. The tissue samples were treated with 3% aqueous hydrogen peroxide to block endogenous peroxidase activity. After being blocked with 3% bovine serum albumin, sections were incubated with primary antibodies overnight at 4 °C. The primary antibodies used for immunohistochemistry were against MCL-1, Ki-67, p-GSK-3β (Ser9), and cleaved caspase 3. The slides were then incubated with secondary antibody. Immunoreactive signals were visualized with a DAB chromogenic substrate kit (Servicebio Technology, cat. No. G1211). Hematoxylin was used for counterstaining.

### Statistical analysis

The results of 3 independent experiments were expressed as mean ± SD as applicable. GraphPad Prism 7.0 software (GraphPad Software, RRID:SCR_002798) was used for statistical analysis. One-way ANOVA (Tukey’s multiple comparisons test) and unpaired Student’s *t*-test were appropriately used to quantify the statistical differences between groups, with a significance level of *P < 0.05.

## Results

### Bilirubin decreases the anticancer activity of sorafenib in HCC

**Figure 1:**
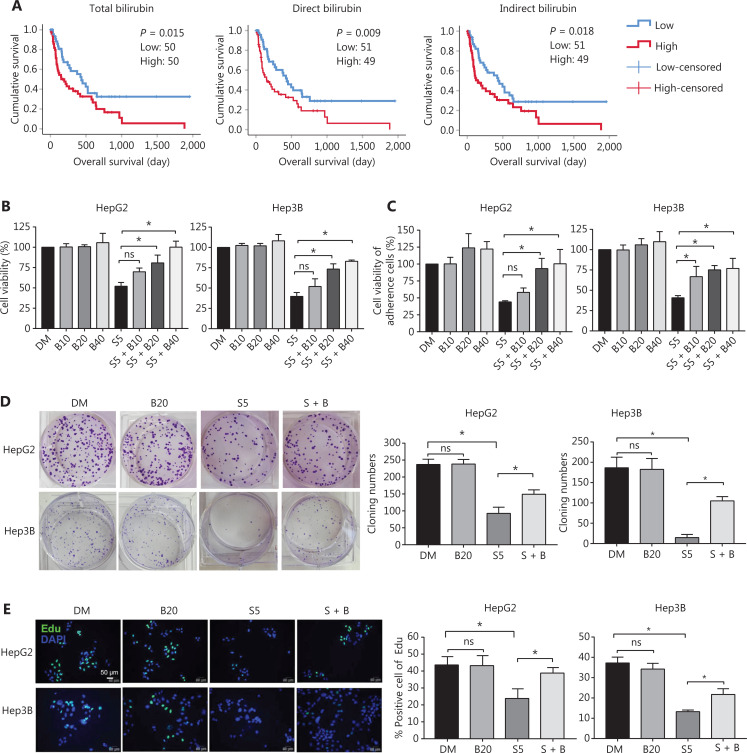
Bilirubin reverts the anti-cancer effect of sorafenib in HCC. (A) Serum bilirubin may predict outcomes of patients with HCC after sorafenib treatment. Kaplan-Meier curves for HCC overall patient survival according to serum bilirubin (total, direct, and indirect) levels are shown. Low bilirubin (≤ median of bilirubin levels); high bilirubin (> median of bilirubin levels). (B) HepG2 and Hep3B cells were treated with 10 μM, 20 μM, or 40 μM bilirubin (B10, B20, or B40) and/or 5 μM sorafenib (S5) for 24 h, and cell viability was measured with MTS assays. Mean ± SD (*n* = 3), *P < 0.05. ns: no significance. (C) HepG2 and Hep3B cells were treated with 10 μM, 20 μM, or 40 μM bilirubin (B10, B20, or B40) and/or 5 μM sorafenib (S5) for 24 h, and the viability of attached cells was measured by crystal violet staining. Data are shown from 3 independent replicates. Mean ± SD (*n* = 3), *P < 0.05. ns: no significance. (D) HepG2 and Hep3B cells were treated with 20 μM bilirubin (B20), 5 μM sorafenib (S5), or their combination (S + B) for 24 h, and cell proliferation was measured by cloning formation assays. Mean ± SD (*n* = 3), *P < 0.05. ns: no significance. (E) HepG2 and Hep3B cells were treated with 20 μM bilirubin (B20) and/or 5 μM sorafenib (S5) for 24 h, and cell proliferation was measured with EdU staining assays. Mean ± SD (*n* = 3), *P < 0.05.

To determine the effect of bilirubin on sorafenib therapy, we studied a retrospective observational cohort of 100 patients and analyzed the relationship between serum bilirubin levels before treatment and the overall survival of patients with HCC treated with sorafenib. The baseline characteristics of all patients and statistical analysis of serum bilirubin levels are shown in **Supplementary Tables S1 and S2**. The Kaplan-Meier survival curves indicated that patients with HCC with high levels of one of the 3 indexes of serum bilirubin (total, direct, and indirect bilirubin) and had poorer overall survival after sorafenib treatment (*P* < 0.05) than those with low levels (**Figure 1A**). Through univariate and multivariable Cox regression analysis, we found that patients with high levels of total bilirubin had a significantly higher risk of HCC mortality (*P* < 0.05) than patients with low levels (**Supplementary Table S3**). These findings suggested that bilirubin may be associated with poor patient survival outcomes after sorafenib treatment.

To investigate the direct effect of bilirubin on the anticancer activity of sorafenib, we treated HCC cell lines (HepG2 and Hep3B) with sorafenib in the absence or presence of bilirubin for 24 h, then performed MTS assays (**Figure 1B**) and crystal violet staining (**Figure 1C**) to determine cell viability. Sorafenib (5 µM) induced significant growth inhibition of HepG2 and Hep3B cells, but this effect was blocked by bilirubin (20 µM and 40 µM) (*P* < 0.05) (**Figure 1B**). Staining with crystal violet dye also indicated that sorafenib-induced growth inhibition was suppressed by bilirubin (20 µM and 40 µM) in HepG2 and Hep3B cells (*P* < 0.05) (**Figure 1C**). In addition to these short-term cell growth assessments, clone formation assays were performed to assess the long-term cell proliferation ability after drug treatment (**Figure 1D**). Compared with sorafenib alone, combined treatment with sorafenib and bilirubin in HepG2 and Hep3B cells resulted in enhanced clone formation (*P* < 0.05) (**Figure 1D**). EdU labeling assays were also used to detect DNA synthesis in proliferating cells. The percentage of EdU-positive cells in the sorafenib plus bilirubin group was significantly higher than that in the sorafenib alone group (*P* < 0.05) (**Figure 1E**). Collectively, these results suggested that bilirubin antagonizes the anticancer activity of sorafenib in HCC cells.

### Bilirubin inhibits sorafenib-induced growth inhibition and apoptosis in HCC cells

**Figure 2:**
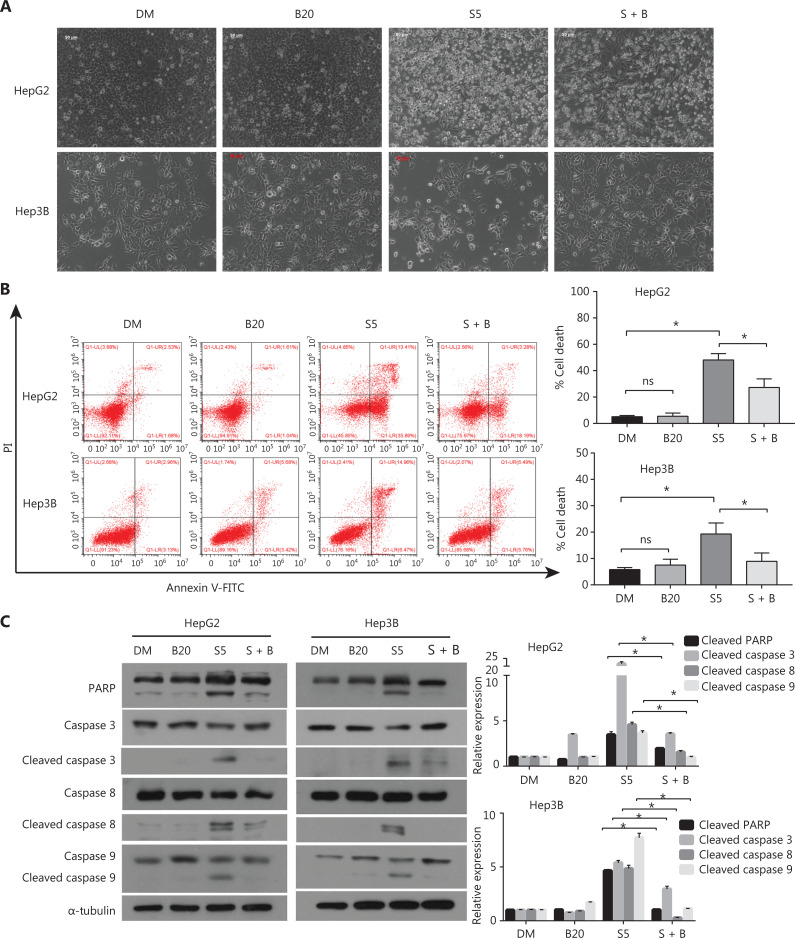
Bilirubin decreases the apoptosis induced by sorafenib in HCC cells. (A) Representative phase-contrast images of HepG2 and Hep3B cells treated with 20 μM bilirubin (B20), 5 μM sorafenib (S5), or their combination (S + B) for 24 h. (B) HepG2 and Hep3B cells were treated with 20 μM bilirubin (B20) and/or 5 μM sorafenib (S5) for 24 h, and cells were then stained with Annexin V-FITC/propidium iodide and analyzed with flow cytometric assays. The relative quantification of cell death is shown. Mean ± SD (*n* = 3), *P < 0.05. ns: no significance. (C) HepG2 and Hep3B cells were treated with 20 μM bilirubin (B20) and/or 5 μM sorafenib (S5) for 24 h. The indicated proteins were analyzed with western blotting. α-tubulin was used as a loading control. Relative quantification of cleavage of PARP and caspase3/8/9 is shown. Mean ± SD (*n* = 3), *P < 0.05. ns: no significance.

Because the induction of apoptotic cell death is an important anti-tumor function of sorafenib^30^, we examined the effect of bilirubin on sorafenib-induced cell death in HCC cells. As shown in **Figure 2A**, sorafenib triggered cell detachment and overt death in HepG2 and Hep3B cells, but this effect decreased after the addition of bilirubin. To further study whether the antagonistic effect of bilirubin against sorafenib was associated with apoptosis inhibition, we performed Annexin V-FITC/PI staining and flow cytometry analysis. The combination of sorafenib and bilirubin in HepG2 and Hep3B cells resulted in less apoptosis than did sorafenib alone (*P* < 0.05) (**Figure 2B**). Apoptosis is a regulated process driven by caspase-mediated proteolytic cleavage cascades. We hypothesized that sorafenib-mediated caspase cleavage might be suppressed by bilirubin. Indeed, as shown in **Figure 2C**. Sorafenib alone induced the cleavage of caspases 3, 8, or 9, but this effect was inhibited in HepG2 and Hep3B cells co-treated with bilirubin (*P* < 0.05). Consistently, bilirubin attenuated sorafenib-induced cleavage of poly (ADP-ribose) polymerase (PARP) (*P* < 0.05), a substrate cleaved by caspase 3 (**Figure 2C**). These findings suggested that bilirubin inhibits sorafenib-induced growth inhibition and apoptosis in HCC cells.

### Bilirubin-mediated inhibition of sorafenib-induced apoptosis involves down-regulation of MCL-1 protein expression

**Figure 3:**
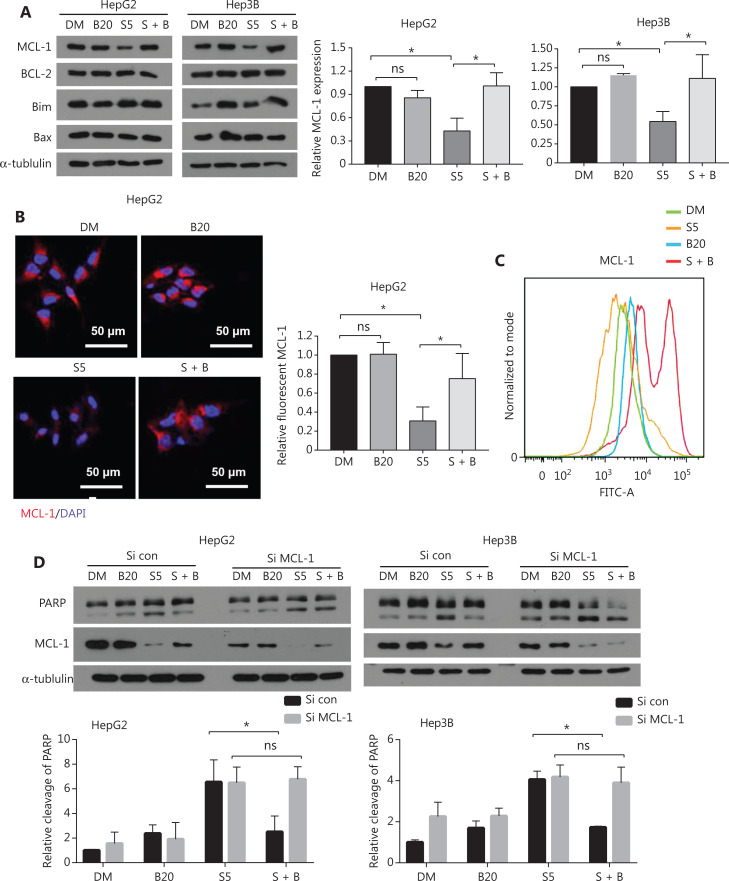
Bilirubin antagonizes sorafenib-mediated downregulation of MCL-1. (A) HepG2 and Hep3B cells were treated with 20 μM bilirubin (B20), 5 μM sorafenib (S5), or their combination (S + B) for 24 h. The indicated proteins were analyzed with Western blot. α-tubulin was used as a loading control. The relative quantification of MCL-1 protein levels is shown. Mean ± SD (*n* = 3), *P < 0.05. ns: no significance. (B) Representative immunofluorescence images showing staining of MCL-1 (red) and DAPI (blue) in HepG2 cells after treatment with 20 μM bilirubin (B20) and/or 5 μM sorafenib (S5) for 10 h. Relative quantification of the fluorescence intensity of MCL-1 is shown. Mean ± SD (*n* = 3), *P < 0.05. ns: no significance. (C) Flow cytometry measurement of MCL-1 expression in HepG2 cells after treatment with 20 μM bilirubin (B20) and/or 5 μM sorafenib (S5) for 10 h. (D) HepG2 and Hep3B cells were transfected with control siRNA or MCL-1 siRNA for 24 h, then treated with 20 μM bilirubin (B20) and/or 5 μM sorafenib (S5) for 24 h. The indicated proteins were analyzed with western blotting. α-tubulin was used as a loading control. Relative quantification of cleavage of PARP is shown. Mean ± SD (*n* = 3), *P < 0.05. ns: no significance.

BCL-2 family members either promote or inhibit apoptosis by controlling mitochondrial outer membrane permeabilization. To explore the molecular mechanism responsible for the antagonistic effect of bilirubin toward sorafenib, we examined expression of the BCL-2 family proteins. Western blot assays revealed that sorafenib decreased the protein level of MCL-1 but not other BCL-2 family proteins (including BCL-2, BIM, and BAX) in HepG2 and Hep3B cells (**Figure 3A**). Notably, the addition of bilirubin prevented sorafenib-induced MCL-1 protein downregulation in HepG2 and Hep3B cells (*P* < 0.05) (**Figure 3A**). Immunofluorescence assays and flow cytometry analysis also revealed that bilirubin inhibited sorafenib-induced MCL-1 downregulation (**Figure 3B, 3C**). To determine the role of MCL-1 protein in the antagonistic effect of bilirubin toward sorafenib, we genetically silenced MCL-1 with a specific siRNA in HepG2 and Hep3B cells. In the control siRNA-treated HepG2 and Hep3B cells, the sorafenib alone group showed greater PARP cleavage than the sorafenib plus bilirubin group (*P* < 0.05) (**Figure 3D**). In contrast, levels of PARP cleavage in the sorafenib alone group were similar to those in the sorafenib plus bilirubin cotreatment group in MCL-1 knockdown HepG2 and Hep3B cells (**Figure 3D**), thus indicating that MCL-1 is required for the antagonistic effect of bilirubin toward sorafenib. Collectively, these data suggested that bilirubin suppresses the apoptotic effect of sorafenib by inhibiting the downregulation of MCL-1 protein.

### Bilirubin inhibits sorafenib-induced MCL-1 degradation

**Figure 4:**
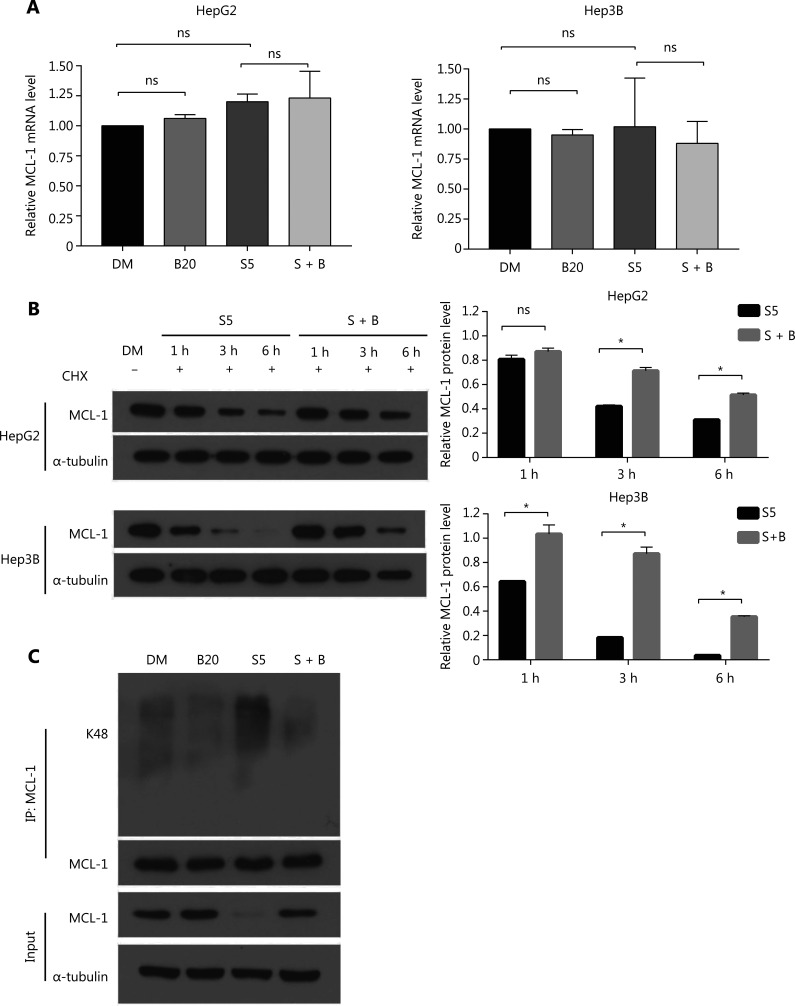
Bilirubin reverses the anti-cancer effect of sorafenib by decreasing MCL-1 degradation. (A) HepG2 and Hep3B cells were treated with 20 μM bilirubin (B20), 5 μM sorafenib (S5), or their combination (S + B) for 6 h, the mRNA expression of MCL-1 was measured by RT-qPCR, and the expression level relative to that of the control was calculated. Mean ± SD (*n* = 3). ns, no significance. (B) HepG2 and Hep3B cells were treated with 5 μM sorafenib (S5), or 5 μM sorafenib and 20 μM bilirubin combination (S + B), with or without cycloheximide (CHX, 25 μg/mL), for the indicated times. MCL-1 was analyzed with western blotting. α-tubulin was used as a loading control. The relative quantification of MCL-1 protein levels is shown. Mean ± SD (*n* = 3), *P < 0.05. ns: no significance. (C) HepG2 and Hep3B cells were treated with 20 μM bilirubin (B20) and/or 5 μM sorafenib (S5) for 24 h, in the presence of the proteasome inhibitor MG132 (10 μM, last 6 h), endogenous MCL-1 was immunoprecipitated, and immunoblotting was performed with antibodies to K48 linked ubiquitin.

Next, we determined whether bilirubin interferes with MCL-1 expression at the transcriptional or post-transcriptional level. RT-qPCR analysis revealed that bilirubin, sorafenib, or their combination did not affect mRNA levels of MCL-1 in HepG2 and Hep3B cells (**Figure 4A**), thus indicating that bilirubin’s blocking of sorafenib-induced MCL-1 down-regulation does not depend on a transcriptional mechanism. MCL-1 is an antiapoptotic protein with a short half-life, which is degraded through the ubiquitin-proteasome system^31,32^. To determine whether MCL-1 protein stability was associated with the antagonistic effect of bilirubin toward sorafenib, we performed cycloheximide chase experiments to determine the half-life of MCL-1 protein. In the presence of CHX treatment, bilirubin still antagonized the sorafenib-mediated decrease in MCL-1 protein expression (**Figure 4B**), thus indicating that bilirubin inhibited the degradation of MCL-1 induced by sorafenib in these cells. Given that K48-linked ubiquitination leads to proteasomal degradation of MCL-1^33^, we examined the effects of sorafenib, bilirubin, and their combination on K48-linked ubiquitination of MCL-1. Sorafenib significantly increased the K48-linked ubiquitination of MCL-1 in HepG2 cells (**Figure 4C**). Importantly, bilirubin completely blocked the accumulation of K48-linked ubiquitination of MCL-1 induced by sorafenib in HepG2 cells (**Figure 4C**). These data suggested that bilirubin suppresses the anticancer effect of sorafenib by inhibiting ubiquitination-mediated MCL-1 degradation.

### Bilirubin inhibits GSK-3β-mediated MCL-1 degradation induced by sorafenib

**Figure 5:**
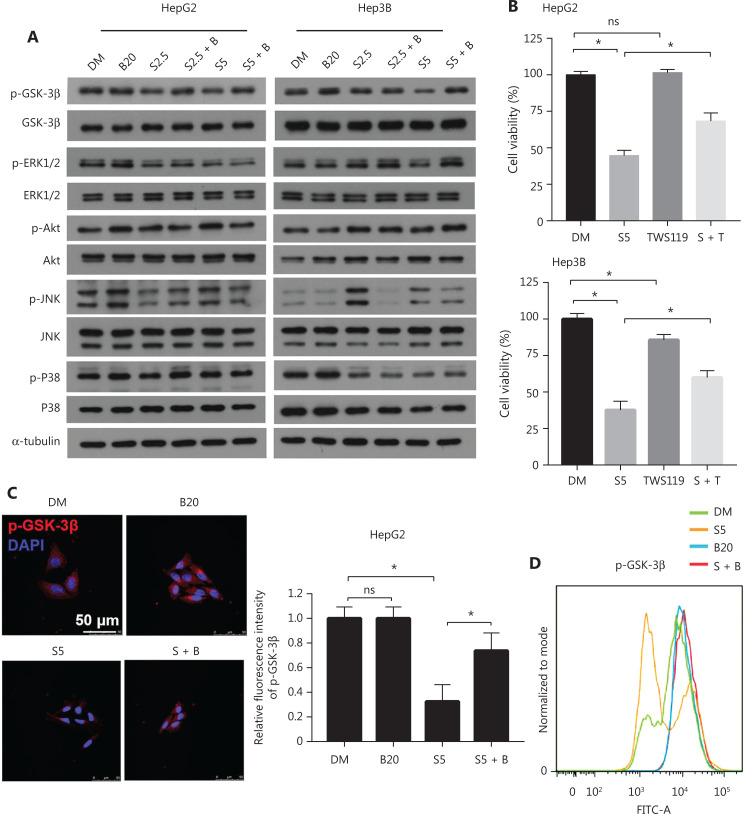
Bilirubin reverses sorafenib induced GSK-3β activation. (A) HepG2 and Hep3B cells were treated with 20 μM bilirubin (B20), 2.5 or 5 μM sorafenib (S2.5 or S5), or their combination (S + B) for 6 h. GSK-3β, ERK1/2, and their phosphorylation were analyzed with western blotting. α-tubulin was used as a loading control. (B) HepG2 and Hep3B cells were treated with 5 μM sorafenib (S5) and/or 2.5 μM TWS119 for 24 h, and cell viability was measured with MTS assays. Mean ± SD (*n* = 3), *P < 0.05. ns: no significance. (C) Representative immunofluorescence images showing staining of p-GSK-3β (red) and DAPI (blue) in HepG2 cells after treatment with bilirubin (20 μM) and/or sorafenib (5 μM) for 6 h. Relative quantification of the fluorescence intensity of p-GSK-3β is shown. Mean ± SD (*n* = 3), *P < 0.05. ns: no significance. (D) Flow cytometry for measurement of p-GSK-3β expression in HepG2 cells after treatment with 20 μM bilirubin (B20) and/or 5 μM sorafenib (S5) for 6 h.

The protein stability of MCL-1 is regulated by its phosphorylation by different kinases. In particular, glycogen synthase kinase 3 beta (GSK-3β) phosphorylates MCL-1 and facilitates its degradation^33^, whereas ERK1/2 phosphorylates MCL-1 and increases its protein stabilization^34^. Therefore, we detected the phosphorylation levels of GSK-3β and ERK1/2. Phosphorylation of GSK-3β at the Ser9 site has been shown to induce its inactivation^35^. We found that sorafenib decreased the phosphorylation of GSK-3β at the Ser9 site in HepG2 and Hep3B cells, thus indicating that sorafenib activates GSK-3β (**Figure 5A**). Of note, this process was inhibited by bilirubin (**Figure 5A**). Consistently, the GSK-3β inhibitor TWS119 attenuated the anticancer effect of sorafenib in HepG2 and Hep 3B cells (*P* < 0.05) (**Figure 5B**), thereby supporting the conclusion that activation of GSK-3β contributes to the anticancer activity of sorafenib. In contrast, sorafenib decreased the phosphorylation of ERK1/2 in HepG2 and Hep3B cells, thus indicating that sorafenib limits the activity of ERK1/2 (**Figure 5A**). However, bilirubin inhibited the sorafenib-induced inhibition of ERK1/2 in only Hep3B cells, but not in HepG2 cells under our experimental conditions (**Figure 5A**). These findings indicated a context-dependent role of ERK1/2 in mediating the effects of bilirubin on sorafenib activity. In addition to Western blot, immunofluorescence staining and flow cytometry analysis confirmed that bilirubin inhibited sorafenib-induced GSK-3β activation (suppression of phosphorylation of GSK-3β at Ser9) in HepG2 cells (**Figure 5C, D**). Together, these findings demonstrated that GSK-3β may play a critical role in regulating bilirubin-mediated inhibition of sorafenib-induced MCL-1 degradation.

Several kinases, including Akt, JNK and p38, act upstream of GSKβ and regulate phosphorylation of GSKβ in human cells^36–39^. Thus, we tested whether bilirubin might inhibit sorafenib-induced GSK-3β activation by affecting the activation of Akt, JNK, and p38. However, no discernible differences were observed in the phosphorylation levels of Akt, JNK and p38 between the sorafenib alone group and the sorafenib plus bilirubin cotreatment group in HepG2 and Hep3B cells (**Figure 5A**). Therefore, we suggest that Akt, JNK, and p38 might not be involved in the antagonistic effect of bilirubin toward sorafenib.

### Bilirubin shows antagonistic effects toward sorafenib *in vivo*

**Figure 6:**
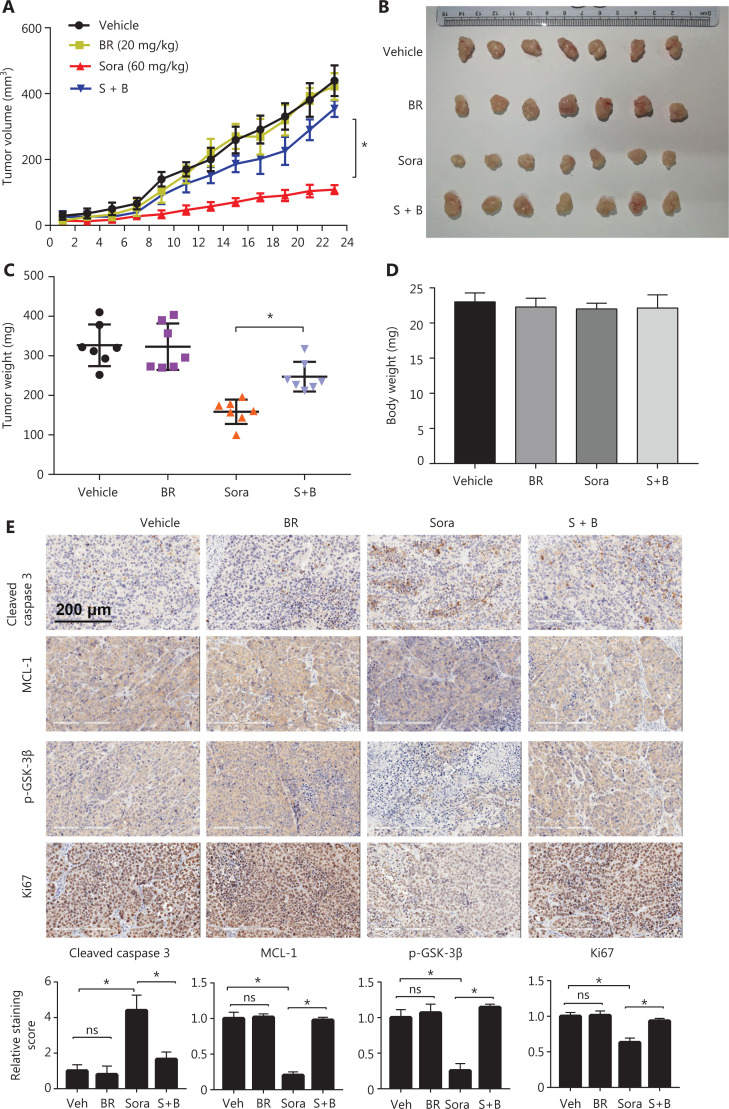
Bilirubin inhibits the anticancer activity of sorafenib *in vivo*. Nude mice were treated with vehicle, bilirubin alone (BR, 20 mg/kg/d), sorafenib alone (Sora, 60 mg/kg/d), or a combination of bilirubin and sorafenib (S + B) for 23 days. (A) Tumor volumes were recorded every 2 days. Mean ± SD (*n* = 7), *P < 0.05. After 23 days of treatment, the mice were sacrificed. Xenograft images (B), tumor weight (C), and body weight (D) are shown. Mean ± SD (*n* = 7), *P < 0.05. (E) Immunohistochemical detection of cleaved caspase 3 (CC3), MCL-1, p-GSK-3β, and Ki-67 in tumor tissues. Relative quantification of the staining intensity of the indicated proteins is shown. Mean ± SD (*n* = 7), *P < 0.05. ns: no significance.

To evaluate the effect of bilirubin on the anticancer activity of sorafenib *in vivo*, we established a xenograft tumor model by subcutaneously inoculating HepG2 cells into nude mice. Compared with the vehicle treatment group, sorafenib treatment inhibited the tumor growth of HepG2 xenografts (**Figure 6A, 6B**). However, bilirubin addition decreased the sorafenib-mediated tumor suppression (*P* < 0.05) (**Figure 6A, 6B**). Consistently, bilirubin significantly inhibited the sorafenib-mediated suppression of the tumor weights of HepG2 xenografts (*P* < 0.05) (**Figure 6C**). No significant difference was observed in body weights among the vehicle, sorafenib, bilirubin, and combination treatment groups (**Figure 6D**), thus indicating that all tested agents were non-toxic to mice. Similarly to the findings in *in vitro* assays, sorafenib decreased the expression of MCL-1 and p-GSK-3β, and increased the expression of cleaved caspase 3 (*P* < 0.05) *in vivo* (**Figure 6E**). However, introduction of bilirubin suppressed the effects of sorafenib in the xenografts (**Figure 6E**). Moreover, bilirubin inhibited the sorafenib-mediated decrease in the percentage of HCC cells positive for Ki-67, a cell proliferation marker (*P* < 0.05) (**Figure 6E**). Overall, these animal studies further supported the conclusion that bilirubin antagonizes the anticancer activity of sorafenib.

## Discussion

**Figure 7:**
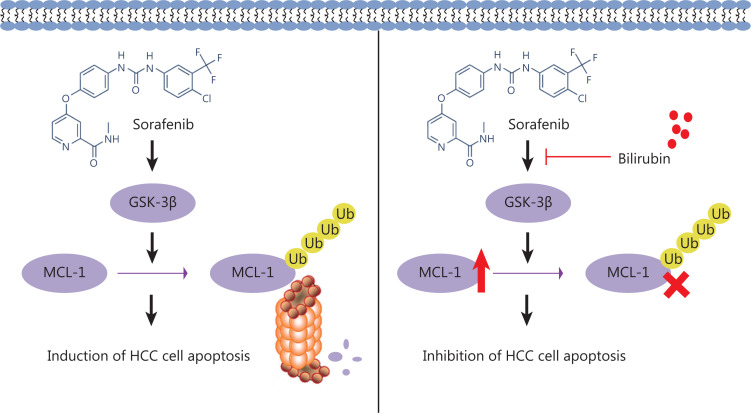
Mechanism of the antagonistic effect of bilirubin on the anti-cancer activity of sorafenib in HCC.

Sorafenib remains the first line systemic treatment for advanced HCC. Although it can improve the survival rate of patients with HCC, its rate of effectiveness remains very low in the long term, owing to the development of drug-resistant cells through multiple mechanisms^8,9^. To overcome sorafenib’s shortcomings, in recent years, researchers have made great efforts to explore specific predictive or prognostic factors in the responsiveness to sorafenib in HCC^40–42^. Among the factors reported, the ALBI grade has received increasing attention, and can be used as a potential criterion for selecting patients for second-line treatment^18,20,21^. However, whether and how bilirubin levels are associated with sorafenib treatment failure are poorly understood. In this study, we investigated the potential involvement of bilirubin in clinical cases of sorafenib failure, explored the relationship between bilirubin and sorafenib in HCC, and discovered the antagonistic effect of bilirubin toward the anti-HCC activity of sorafenib *in vitro* and *in vivo*. Mechanically, sorafenib induces HCC cell apoptosis through activation of GSK-3β and subsequent proteasomal degradation of MCL-1, but these effects are inhibited by bilirubin (**Figure 7**).

Bilirubin, the final product of heme catabolism, has multiple physiological and pathological effects^43,44^. Bilirubin can have positive or negative roles in different pathophysiological processes. For example, it has a protective role in atherosclerosis, but it can cause nervous system damage^45,46^. In the current study, we found that bilirubin has antagonistic effects on the proliferation inhibition and apoptosis-inducing activity of sorafenib. The effects of bilirubin on the anticancer effects of sorafenib were determined with cell viability, proliferation, and apoptosis assays. Compared with sorafenib treatment alone, sorafenib and bilirubin combination treatment resulted in less inhibition of cell viability and proliferation according to MTS assays and crystal violet staining, thus indicating that bilirubin blocks the sorafenib-mediated suppression of cell proliferation. The results of cloning formation experiments and Edu staining further confirmed the antagonistic effect of bilirubin toward sorafenib in HCC cells. Flow cytometry analysis indicated that bilirubin decreased sorafenib-induced apoptosis in HCC cells. Moreover, compared with sorafenib treatment alone, the combination treatment resulted in less activation of apoptosis-related proteins, including the cleavage of PARP, caspase 3, caspase 8, and caspase 9. Further study revealed the antagonistic effects of bilirubin toward the anticancer effects of sorafenib in xenograft models. These findings revealed a new role of bilirubin in determining sorafenib failure in patients with HCC. In the future, bilirubin reduction strategies, such as hemoperfusion, may improve the efficacy of sorafenib in HCC.

BCL-2 family proteins are classified into 2 subgroups: antiapoptotic and proapoptotic proteins. MCL-1 is a major antiapoptotic BCL-2 family member, which plays an important role in tumor development and therapy resistance^47^. Previous studies have shown that the cyclin dependent kinase inhibitor flavopiridol enhances the apoptosis-inducing effects of sorafenib on HCC cells through MCL-1 suppression^48^. In addition, Ras association domain family member 6 (RASSF6) increases sorafenib-mediated apoptosis in renal cell carcinoma by repressing MCL-1 in a c-Jun N-terminal kinase (JNK)-dependent manner^49^. These findings highlight that the regulation of MCL-1 affects the sensitivity of cancer cells to sorafenib. In this study, the knockdown of MCL-1 inhibited the antagonistic effects of bilirubin toward sorafenib-induced apoptosis, thus indicating that MCL-1 plays a key role in determining sorafenib sensitivity. Sorafenib also induces autophagy-dependent cell death through the activation of beclin 1 mediated by MCL-1 in HCC cells^50^. Alternatively, sorafenib’s anticancer activity is mediated by inhibiting system xc^-^ in HCC cells, thereby inducing GSH depletion and subsequent ferroptosis^51^. These findings indicate that sorafenib may induce different types of cell death, depending on the concentration and duration of treatment. Whether bilirubin also affects ferroptotic and autophagic cell death remains to be investigated. Regardless, we suggest that bilirubin levels should be monitored routinely during sorafenib treatment in clinical settings.

MCL-1 is degraded though the ubiquitin-proteasome pathway^52^ or autophagy pathway^53^. In this study, CHX chase experiments indicated that the half-life of MCL-1 protein in HCC cells was longer in the bilirubin and sorafenib co-treatment group than in the group treated with sorafenib alone. We further observed that the ubiquitination of MCL-1 was upregulated by sorafenib, and this process decreased with bilirubin addition. Therefore, bilirubin may inhibit the degradation of MCL-1 through the ubiquitination-mediated proteasome pathway. Determining the direct components of the ubiquitin-proteasome pathway responsible for bilirubin-regulated degradation of MCL-1 will be important.

The stability of MCL-1 protein involves phosphorylation of MCL-1 at different sites^52^. For instance, ERK1/2 phosphorylates MCL-1 at the Thr163 site and increases the half-life of MCL-1, thereby enhancing its anti-apoptotic function^34^. In contrast, GSK-3β phosphorylates MCL-1 at the Ser159 site, thus facilitating ubiquitination and degradation of MCL-1^33^. GSK-3β has been shown to act as a negative regulator of ERK1/2 in colon cancer cells^54^, thus indicating the feedback mechanisms of different signaling pathways. We found that sorafenib induced the activation of p-GSK-3β and the inhibition of ERK1/2. However, our results indicated that bilirubin plays a broad role in regulating GSK-3β phosphorylation (but not ERK1/2 phosphorylation) in both Hep3B and HepG2 cells. Consequently, GSK-3β is likely to play a key role in bilirubin-mediated regulation of MCL-1 degradation. Bilirubin may therefore be crucial in the inactivation of GSK-3β that stabilizes MCL-1 protein and ultimately weakens the anticancer ability of sorafenib. However, this study has a limitation in that how bilirubin inhibits GSK-3β phosphorylation remains unknown. Our findings only suggest that Akt, JNK, and p38, known kinases acting upstream of GSK-3β, may not be involved in the antagonistic effect of bilirubin toward sorafenib (**Figure 5A**). In addition to direct binding to GSK-3β, possible mechanisms include bilirubin-mediated inhibition of protein kinase A^55^ and protein kinase C^56^, which may also be involved in the upstream pathway of GSK-3β phosphorylation. These hypotheses must be examined in future research.

## Conclusions

In summary, we demonstrate that bilirubin inhibits the activity of sorafenib in HCC cells by blocking MCL-1 degradation in a GSK-3β-dependent manner. These new findings may provide a potential strategy to overcome the clinical failure of sorafenib.
