# Supplementary material for: Bilirubin inhibits the anticancer activity of sorafenib by blocking MCL-1 degradation in hepatocellular carcinoma cells
Source: Cancer Biol Med. 2022 May 24;19(7):1061–77. doi: 10.20892/j.issn.2095-3941.2021.0598 (PMC9334754; doi:10.20892/j.issn.2095-3941.2021.0598)
Supplement: Supplementary file 1 [file cbm-19-1061-s001.pdf]

Supplementary materials

Table S1 Baseline characteristics of cohort patients according to bilirubin level

	TBIL			DBIL			IBIL		
	Low	High	*P* value	Low	High	*P*	Low	High	*P* value
Gender (%)			1.000			0.941			0.941
Female	6 (50.0)	6 (50.0)		6 (50.0)	6 (50.0)		6 (50.0)	6 (50.0)	
Male	44 (50.0)	44 (50.0)		45 (51.1)	43 (48.9)		45 (51.1)	43 (48.9)	
Age (years, mean ± SD)	50.22 ± 11.94	47.72 ± 12.08	0.300	48.67 ± 12.15	49.29 ± 11.99	0.798	52.63 ± 10.85	45.16 ± 12.08	0.002
HBV (%)	49 (49.5)	50 (50.5)	1.000	50 (50.5)	49 (49.5)	1	50 (50.5)	49 (49.5)	1
Alcohol drinkers (%)	10 (55.6)	8 (44.4)	0.603	11 (61.1)	7 (38.9)	0.343	8 (44.4)	10 (55.6)	0.539
Portal vein or inferior vena cava invasion	24(44.4)	30(55.6)	0.229	24(44.4)	30(55.6)	0.229	24(44.4)	30(55.6)	0.229
EM* (%)			0.056			0.173			0.731
0	27 (43.5)	35 (56.5)		29(46.8)	33(53.2)		31 (50.0)	31 (50.0)	
1	11 (55.0)	9 (45.0)		12(60.0)	8(40.0)		9 (45.0)	11 (55.0)	
2	8 (88.9)	1 (11.1)		7(77.8)	2(22.2)		6 (66.7)	3 (33.3)	
3	4 (44.4)	5 (55.6)		3(33.3)	6(66.7)		5 (55.6)	4 (44.4)	
Ascites (%)			0.081			0.012			0.332
None	36(59.0)	25(41.0)		39 (63.9)	22 (36.1)		35 (57.4)	26 (42.6)	
Small	8(34.8)	15(65.2)		7 (30.4)	16 (69.6)		9 (39.1)	14 (60.9)	
Moderate	3(27.3)	8(72.7)		3 (27.3)	8 (72.7)		4 (36.4)	7 (63.6)	
Large	3(60.0)	2(40)		2 (40.0)	3 (60.0)		3 (60.0)	2 (40.0)	
Smokers (%)	17 (43.6)	22 (56.4)	0.305	18 (46.2)	21 (53.8)	0.438	19 (48.7)	20 (51.3)	0.715
Child-Pugh (%)			< 0.001			< 0.001			0.001
A	35 (72.9)	13 (27.1)		38 (79.2)	10 (20.8)		32 (66.7)	16 (33.3)	
B	14 (33.3)	28 (66.7)		12 (28.6)	30 (71.4)		18 (42.9)	24 (57.1)	
C	1 (10.0)	9 (90.0)		1 (10.0)	9 (90.0)		1 (10.0)	9 (90.0)	

Table S1 Continued

	TBIL			DBIL			IBIL		
	Low	High	*P* value	Low	High	*P*	Low	High	*P* value
BCLC (%)			0.478			0.487			0.309
A	0 (0)	1 (100)		0(0)	1 (100.0)		1 (100)	0(0)	
B	16 (48.5)	17 (51.5)		17(51.5)	16(48.5)		19(57.6)	14(42.4)	
C	34 (51.5)	32 (48.5)		34(51.5)	32(48.5)		31(47.0)	35(53.0)	
HCC (%)			0.002			0.002			0.009
Initial HCC	48 (56.5)	37 (43.5)		49(57.6)	36(42.4)		48 (56.5)	37 (43.5)	
Recurrent HCC	2 (13.3)	13 (86.7)		2(13.3)	13(86.7)		3 (20.0)	12 (80.0)	
TBIL (μmol/L, median ± IR)	11.55 ± 5.20	47.40 ± 67.70	< 0.001	11.60 ± 5.40	49.50 ± 67.50	< 0.001	11.70 ± 5.80	44.0 ± 72.8	< 0.001
DBIL (μmol/L, median ± IR)	5.25 ± 2.7	27.10 ± 44.80	< 0.001	5.20 ± 2.60	27.10 ± 46.7	< 0.001	5.40 ± 4.80	23.80 ± 51.7	< 0.001
IBIL (μmol/L, median ± IR)	6.35 ± 3.0	19.10 ± 17.20	< 0.001	6.7 ± 4.20	18.65 ± 19.0	< 0.001	6.50 ± 3.30	20.2 ± 16.80	< 0.001
AFP (ng/mL, median ± IR)	392 ± 7669	773.50 ± 22464	0.325	609.8 ± 9312	487.52 ± 16773	0.743	241 ± 2781	1201 ± 38840	0.004
ALT (median ± IR)	33.50 ± 32.0	76 ± 72	0.001	34 ± 35	76 ± 66	0.001	35.0 ± 35	76 ± 63	0.002
AST (median ± IR)	48.50 ± 48	179 ± 208	< 0.001	45 ± 52	165.5 ± 181	< 0.001	52.5 ± 51	179 ± 208	< 0.001

Table S2 Statistical analysis of serum bilirubin levels

	n	Minimum	Maximum	Mean	Median	IR
TBIL	100	5	300.4	42.27	19.95	35.9
DBIL	100	1.8	173.6	25	9.8	21.3
IBIL	100	1.2	133.1	17.19	10.2	13.4

Table S3 Cox regression analysis of characteristics associated with overall survival time

Baseline covariate	Univariate model		*P* value	Multivariate model		*P* value
	HR	95%CI		HR	95%CI	
Smoking (yes *vs.* no)	2.349	1.412–3.908	0.001	2.648	1.571–4.461	< 0.001
Ascites (yes *vs.* no)	3.079	1.820–5.212	< 0.001	2.286	1.304–4.009	0.004
Child Pugh (B *vs.* A)	1.883	1.097–3.232	0.022			
Child Pugh (C *vs.* A)	3.036	1.401–6.577	0.005			
BCLC (C *vs.* B)	2.47	1.373–4.445	0.003			
EM (yes *vs.* no)	1.702	1.023–2.834	0.041	1.693	1.012–2.835	0.045
TBIL	1.01	1.006–1.014	< 0.001	1.008	1.004–1.013	< 0.001
DBIL	1.014	1.008–1.020	< 0.001			
IBIL	1.023	1.013–1.033	< 0.002			
AST	1.001	1.0002–1.002	0.016			
